# A framework for the responsible reform of the 14-day rule in human embryo research

**DOI:** 10.1007/s13238-022-00907-5

**Published:** 2022-02-15

**Authors:** Yaojin Peng, Jianwei Lv, Zhenyu Xiao, Lulu Ding, Qi Zhou

**Affiliations:** 1 State Key Laboratory of Stem Cell and Reproductive Biology, Institute of Zoology, Chinese Academy of Sciences, Beijing, 100101 China; 2 Institute for Stem Cell and Regeneration, Chinese Academy of Sciences, Beijing, 100101 China; 3 University of the Chinese Academy of Sciences, Beijing, 100049 China; 4 Beijing Institute for Stem Cell and Regenerative Medicine, Beijing, 100101 China

Ever since the world’s first “test-tube” baby was born in 1978, the use of human embryos in research has become highly controversial ethically and thus created a significant challenge for jurisdictions, especially in Europe and the United States, to determine the relevant policies (Poplawski and Gillett, 1991). To resolve the dispute and responsibly promote human embryonic research, the 14-day rule, which prohibits culturing human embryos *in vitro* beyond 14 days or the onset of primitive streak, was proposed over 40 years ago (Hyun et al., 2016). This rule has become a widely accepted bioethical norm and has been introduced into laws or guidelines by many jurisdictions, such as the United Kingdom, Australia, and China (Matthews et al., 2020). However, recent scientific advancements and policy debates have put this rule under increasing strain. Particularly, the major revision to the 14-day rule proposed by the International Society for Stem Cell Research (ISSCR) latest updated guidelines has fuelled heated debates and raised concerns worldwide (International Society for Stem Cell Research, 2021).

For China, whose scientific concerns, traditional culture, and religion are largely different from the West, the debates and challenges are different and somewhat unique. Thus, it is essential to present a responsible and coordinated way, suitable for Chinese ethical and cultural values and public acceptance, to cope with the potential adjustment of the 14-day rule and to promote the development of the entire embryo research endeavor in China. We first explore recent advances in embryonic and pertinent research fields that directly or indirectly challenge the 14-day rule. Within this context, we then evaluate the overall ethical controversies worldwide and the recent public debate on the 14-day rule in China. Separately, we discuss China’s regulatory framework for human embryo research, including the 14-day rule. On this basis, we lastly provide an essential coordinated framework and strategies to deal with the possible changes to the 14-day rule based on China-specific conditions. We hope that this discussion helps both China and other countries establish a framework of mutual respect, win-win cooperation, and responsibility for future embryo research.

## THE TECHNOLOGY

In the past few years, the fast development of extended *in vitro* culture systems for human, non-human primates, and mouse embryos have provided illuminating insights into early embryonic development from implantation to early organogenesis. In 2016, two studies demonstrated growing human embryos in the lab for 13 days after fertilization, and further culturing has been suspended based on ethical issues rather than technological infeasibility (Deglincerti et al., 2016; Shahbazi et al., 2016), which raised valid concerns regarding the 14-day rule in hindering the development of embryonic research (Regalado, 2021). Moreover, the reports of culturing non-human primate embryos *in vitro* up to 20 days after fertilization and of a platform to support *in vitro* cultured mouse embryos to early organogenesis stages, in 2019 and 2020 respectively (Ma et al., 2019; Niu et al., 2019; Gu et al., 2020), have raised technological feasibility to grow human embryos in the lab beyond 14 days using methods adapted from their work if the 14-day rule does not stand in the way. This has further unleashed impressive pressure on the review of the 14-day rule.

Noteworthily, advancements on *in vitro* culture of non-human primate embryos and stem cell-based embryonic models have indirectly raised another considerable challenge for the 14-day rule. Since this kind of models has been created to replace the research on natural human embryos (Sozen et al., 2021; Weatherbee et al., 2021), the authenticity of these models still needs a benchmark through an assessment based either on *in vitro* human embryo culture systems or *in vivo* counterparts. However, *in vivo* study of embryos is still a daunting task ever since the embryo is implanted into the uterus. Thus, *in vitro* human embryo research beyond 14 days holds the key for the “black box” event that follows gastrulation (Hyun et al., 2021).

## ETHICAL ISSUES AND PUBLIC DEBATES

The debate over embryo research primarily centered on the moral status of human embryos. Diverse moral grounds, especially influenced by different religions, have led to different views regarding the moral status of embryos, ranging from the full status as human beings to none at all (Department of Health and Social Security, 1984). Defenders, for instance, influenced by Catholic doctrines, hold the full moral status view that the embryo becomes a human being or acquires full personhood the same as a grown person from the moment of fertilization (Doerflinger, 1999). By contrast, some believe that an embryo is just a cluster of cells and has no status as a human being (Reichlin, 1997; Brown, 2007). However, the majority are somewhere in between and maintain that the embryo has some intrinsic value; they believe that the moral status of the embryo gradually increases from fertilization to birth. In a way, the Chinese public holds the intermediate view and believes that a human embryo should be respected and protected but can also be used in research (Peng et al., 2020).

The debate on the extension of the 14-day rule should have centered on the ethical status of embryos. However, as the Warnock Report indicated, it seems complicated to reconcile the views and decide which one should prevail (Department of Health and Social Security, 1984). Thus, supporters for the extension attempt to avoid falling into the debate on the ethical status of embryos per se (Appleby et al., 2018; Chan, 2018; Hyun et al., 2021) and continue the utilitarian thought grounded in scientific benefits of allowing human embryos to be cultured beyond 14 days (Hurlbut et al., 2017; McCully, 2021), such as uncovering mechanisms underlying early development in both health and disease and examining the effect of exogenous factors on gastrulating embryos. The opponents argue that such an extension not only fails to respond positively to the moral status of embryos but may also lead to a slippery slope (Blackshaw and Rodger, 2021).

Regarding the 14-day rule per se and the extension, so far, there has been minimal public debate in China. According to the big data public opinion analysis platform of Sina Yuqingtong (https://yqt.mdata.net/), from May 27, 2021, to August 18, 2021 (a period after the ISSCR released the updated guidelines), the total number of discussions on the topic of the 14-day rule is only 135, mainly on Weibo, News App, WeChat, and other platforms (See Table 1). It is evident that the proposal of changing the 14-day rule in the ISSCR updated guidelines has not attracted much attention from the Chinese public. But we can still find some information from the limited discussion that the Chinese public generally is not strongly opposed to the extension of 14-day rule. To be specific, the overall attitude of the debate is relatively neutral (*n* = 61; 46.21%) and supportive attitude (*n* = 41; 31.82%). Only 11.36% (*n* = 15) of the discussions opposed extending the 14-day rule.

**Table 1:** Discussions on the topic of the 14-day rule in China

Total (pieces)	Attitude	Amount of discussion (pieces)	Proportion (%)
135 Source: Weibo WeChat Tencent news	Neutral	61	46.21
	Pros	41	31.82
	Cons	15	11.36

## THE 14-DAY RULE AND BEYOND

In fact, when western countries launched ethical and policy debates around embryo research due to *in vitro* fertilization in the 1980s and embryonic stem cell research around 2000, embryo research did not cause much controversy in China. Despite this, China still introduced regulatory rules concerning embryonic research, including the 14-day rule, in the Ethical Guidelines for Human Embryonic Stem Cell Research (hereafter Ethical Guidelines) in 2003. The primary reason behind such introduction was not deeply rooted in China’s unique ethical culture, but to respond to relevant international concerns (Zhang, 2012). In any case, the 14-day rule and other relevant rules prescribed in the Ethical Guidelines not only have played an essential role in the governance of embryonic and stem cell research at home, but also promoted the communication of biotechnology research and ethical governance among China and other countries in the world in the past ten years.

**Figure 1:**
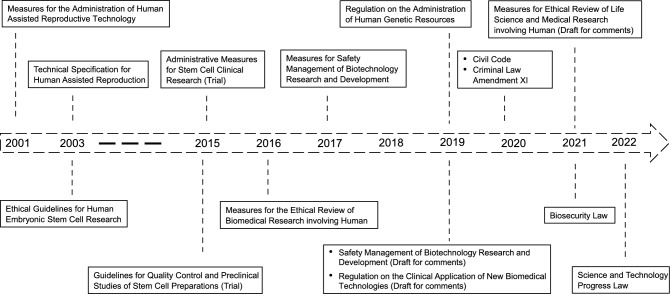
**Normative legal documents regarding human embryo research in China**

Recently, China has been paying increasing attention to ethical governance in science and technology and accelerating the construction of its legislative regime in biotechnology (Fig. 1). For instance, by issuing the Civil Code (2020), Criminal Law Amendment XI (2020), and Biosecurity Law (2021), revising the Science and Technology Progress Law (2022), and drafting the Regulation on Safety Management of Biotechnology Research and Development, the Regulation on the Clinical Application of New Biomedical Technologies, China has already established a relatively systematic biomedical legal system. Moreover, it is noteworthy that in 2019 China set up the National Science and Technology Ethics Committee (NSTEC) to strengthen the governance of scientific and technological ethics.

However, China is still facing legislative and regulatory challenges in the field of embryonic research. Besides the 14-day rule, for instance, under China’s current legal system, the definition of “human embryo” is unclear. Although the Civil Code explicitly provides that a human embryo can be used in research, there is no specific definition of the embryo. In addition, the question of whether stem cell-based models and chimera embryos should be regulated under the regulatory framework of embryo research is waiting for further clarification. Thus, it is necessary for China to systematically reform or improve the 14-day rule and relevant regulatory documents to promote the establishment of a more effective biotechnology regulatory system.

## THE FUTURE

**Figure 2:**
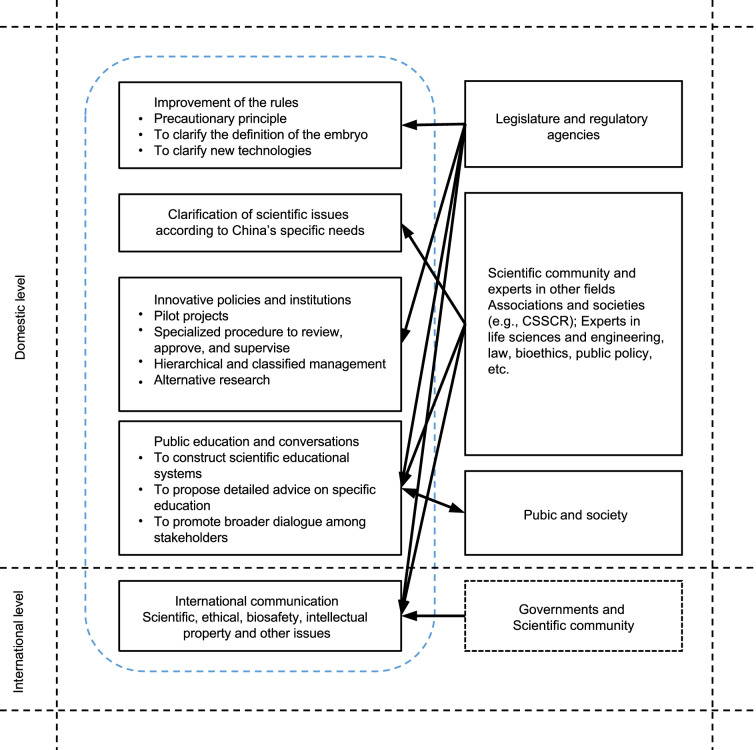
**Responsible framework and strategies for reforming the 14-day rule in China**

Considering the importance of embryonic and pertinent research, the possibility of international policy adjustment, and the recent reform of science and technology ethical governance, it is time for China to re-examine the 14-day rule. From the discussions above, we can conclude that extending the 14-day rule, to a certain extent, will not evoke intense repercussions from the Chinese public. Nevertheless, a comprehensive, prudent, and stepwise approach (Fig. 2) to promote the revision and improvement of China’s legislation and policies will help this country ensure the social acceptance of human embryonic research at home and gain more support and trust from the international community in the long term.

First, if China intends to revise the 14-day rule, the fundamentally effective way is to comprehensiv.ely re-examine and improve the rules for embryonic and related research. The precautionary principle, enshrined in China’s Biosecurity Law (Article 3), should be one fundamental principle to be applied to reflect on any improvement of the rules as well as any review and approval of embryonic and related research. Since it is still unclear what a human embryo is under China’s current legal framework, clarifying the definition of the embryo while reforming the 14-day rule would be a fundamental element. In addition, drawing a new specific line to replace the 14-day limit for proper embryo research is also an important consideration, as it helps to provide more detailed and clear guidance to scientists and regulators. In the meantime, it is time for China to consider the clarification of regulating new technologies, such as research on “admixed” embryos, stem cell-based embryonic models, and three-dimension bioprinting. Noteworthily, it would be appropriate for China to continue to make relevant provisions on these issues in the form of guidelines (e.g., the Ethical Guidelines). Unlike laws or regulations, the guidelines in China present a certain degree of binding force and, at the same time, are more plastic to be revised than laws and regulations in adapting to the rapid advance of life science and technology.

Second, it is imperative to clarify scientific issues according to China’s specific needs, especially local significance. As stated in the ISSCR latest updated Guidelines, only based on the accurate judgments on the critical scientific problems to be solved beyond 14 days as well as their importance and necessity can we further decide how to extend. However, the ISSCR Guidelines do not consider a crucial issue: although the scientific community is generally curious about the unexplored developmental events, from gastrulation onward to the stages when abortus materials are available, due to different social, cultural traditions, population health, demographic and natural environment, the layout of raised basic scientific questions and clinical benefits raised by the extension of the 14-day rule may vary widely by country and region. For instance, probably due to diet or folic acid supplements, the incidence of perinatal neural tube defects varies among European countries (Wadman, 2021). In China, recently, the incidence of perinatal neural tube defects has considerably decreased due to a series of programs related to the prevention and control of birth defects, such as distributing folic acid to pregnant women, but that of congenital heart defects and oral facial clefts has not (Li and Di, 2021). Moreover, with the implementation of the three-child policy (announced on May 31, 2021) in China, the proportion of birth to women with advanced maternal age (> 35) will increase and, thus, the prevalence of congenital anomalies may rise. In this regard, one of the key concerns of China’s biomedical research, especially the research on embryos beyond 14 days, should probably focus on dissecting the etiology of these specific and increasingly severe congenital anomalies in this country. In the meantime, a clear distinction must be drawn between which scientific issues can be solved by embryos research within the 14-day period or alternative research and which can only be addressed by using embryos research beyond 14 days.

The above-mentioned discussions can be initiated by scientific associations and societies related to embryo research in China, such as the Chinese Society for Stem Cell Research (CSSCR). The government may provide infrastructural support and promote intellectual cross-disciplinary interactions through running research fellowships, studentships, seminars, workshops, etc. On this basis, it is vital to seek more extensive expert opinions and strive to reach a basic scientific consensus.

Third, while revisiting and modifying the rules related to embryo research, China could formulate more innovative policies and institutions, with adequate and institutionalized processes of concurrent evaluation. For instance, we recommend that a pilot project be launched to allow a small number of laboratories with specific qualifications and conditions to conduct such research at first. At the same time, for these projects, China may consider establishing a specialized, strict procedure to review, approve, and supervise the *in vitro* embryo research beyond 14 days of these specific laboratories to deter drifts. The procedure should include a scientific peer-review process and an independent specialized ethics review and oversight process (Hyun et al., 2021). More importantly, in this procedure, the ethics committee should be composed of scientists, ethicists, legal and regulatory experts, and community members familiar with embryo research.

In addition, China may consider hierarchical and classified management for *in vitro* embryo research beyond 14 days. For instance, abnormal embryos, such as embryos with gene defects and triploid embryos, which do not have the capability of developing into whole human beings, can be allowed to be used for such research first, and then normal human embryos. In addition, the development of alternative ways, including the use of artificial intelligence simulations, device simulations, animal experiments, embryoids, to reduce the use of human embryos, including research beyond 14 days, ultimately to replace unnecessary human embryo experiments, is also something worth advocating in this country. Only when no valid alternative approach to obtaining the same information exists can embryo research beyond 14 days be allowed.

Fourth, regulators, scientists, and educators are recommended to place more emphasis on public education and conversations to allow for broad engagement and public trust regarding the future expansion of the 14-day rule. As analyzed above, the relevant public debate in China is limited, and embryo research is complicated and ethically controversial worldwide. Considering complex issues concerning ethics and policy, especially in the West, constructing serious and rigorous educational systems of disseminating adequate, evidence-based, timely and culturally pertinent information regarding embryonic research in China would also be helpful to promote scientists’ and the public’s understanding of domestic and international concerns. More importantly, it is vital for scientists and educators, based on China’s specific conditions, to propose detailed advice on the content of the education, the instructional approaches, and the expected goal of the education for the public. This will be an excellent opportunity to raise Chinese public awareness and understanding of embryo research in general and its related research, such as stem cell-based embryo models and chimera research.

In addition to public education, efforts should also be made to promote broader dialogue among multiple stakeholders, including policymakers, scientists, the public, patients, embryo donors, and funders. The dialogue should focus on broader issues, including the scientific, ethical, legal, ethical, and policy issues, raised by permitting such research. In China, we believe that it is more beneficial and feasible for the dialogue to be initiated by academic institutions and research entities from a small scale to a large scale. Stakeholders, such as embryo donors and patients, are encouraged to be involved in the communication in the form of conferences, seminars, or workshops attended by multidisciplinary experts to further promote understanding and trust among different stakeholder groups and contribute to the implementation of relevant policies.

Fifth, it would be necessary for China to participate actively in discussing relevant international policies and strengthen international communication based on value pluralism. The latest ISSCR updated guidelines do not define a clear boundary for human embryo research. It can be foreseen that it is unlikely to reach an agreement around the world on limits or criteria for the change of the 14-day rule in a short time, and various policies will highly possibly be developed in the future, due to the influence of social, cultural, religious, and other factors. Despite this, international conversations and regulatory cooperation are still crucial, particularly considering the responsible global governance of biotechnology.

In promoting the revision of domestic legislation and policies, countries, including China, need to remain transparent and maintain high-quality international dialogue, to promote the trust and support of the international community. Ongoing international dialogue should focus on scientific, ethical, biosafety, intellectual property and other issues concerning embryonic research, particularly regarding the potential impact of extending the 14-day rule on the international community and society. For instance, in the matter of the intellectual property issues, given that the patentability of inventions related to embryo research varies globally (Cuchiara et al., 2013), researchers from the jurisdiction that first extend the 14-day rule will have more opportunities to gain the advantage of developing global patent portfolio. This requires legislators and policymakers worldwide to fully consider its far-reaching impact in the field of biomedicine pertinent to embryonic research. Thus, countries, at least biotechnology developed countries, should try their best to reach an agreement on the revision of the 14-day rule through communication and consultation. Perhaps if several countries intend to revise the 14-day rule, it would be better for them to have a certain degree of synchronization in the time of modifying the rule. Doing so can promote the realization of effective global governance of life technology and avoid competing countries falling into the “slippery slope” of regulation due to factors such as patent considerations.

## CONCLUSIONS

The breakthrough of embryo and related research in the global frontier has rekindled relevant ethical controversies and, directly or indirectly, posed a fresh set of challenges to the 14-day rule. The debate on the extension of the 14-day rule virtually falls within the broader discussion on the ethical and legal implications of human embryo research. However, in China, the relevant public debate is limited, probably due to the intermediate view regarding the moral status of embryos held by the public. Based on this, the potential revision of the rule in China is unlikely to encounter large-scale public opposition. Despite this, in the long run, to support innovation, promote public confidence and ensure mutual-trust and understanding in the international community, a responsible and stepwise reform of the 14-day rule in China is considerably crucial. While revisiting the rule, China should seize this opportunity to carefully re-examine and improve embryo related legislation and policies, especially in the context of the rapid advance of technology in recent years. Moreover, China needs to clarify scientific issues, particularly in accordance with its specific scientific needs, and formulate more innovative policies and institutions, such as establishing specific procedures and launching pilot projects. We believe that although there is little public controversy, China should still pay attention to its public education and dialogue to promote public understanding of relevant research and public confidence in the regulatory process. More importantly, when revising domestic legislation and policies, China should also actively participate in and promote the international exchanges to obtain more support and understanding from the international community. In this way, the potential reform of the 14-day rule in China not only conforms to the ethical culture at home, but also respects the extensive attention from abroad, and ultimately promotes responsible innovation in the field of global embryo research.

## DECLARATIONS

Yaojin Peng, Jianwei Lv and Zhenyu Xiao should be considered joint first author.

We would like to thank Dr. Hongmei Wang, Baoyang Hu, Wei Li, Tongbiao Zhao, Shiqiang Huang, and Haoyi Wang (Chinese Academy of Sciences) for their invaluable comments and advice. This work was supported by the Chinese Academy of Sciences, Grant/Award Number: XDA16040503, the Ministry of Science and Technology, Grant/Award Number: 2018YFE0204403 and the Youth Innovation Promotion Association of the Chinese Academy of Sciences, Grant/Award Number: 2021080.

## References

[CR1] Appleby JB, Bredenoord AL (2018). Should the 14-day rule for embryo research become the 28-day rule?. EMBO Mol Med.

[CR2] Blackshaw BP (2021). J Med Ethics.

[CR3] Brown MT (2007). The potential of the human embryo. J Med Philos.

[CR4] Chan S (2018). How and why to replace the 14-day rule. Curr Stem Cell Rep.

[CR5] Cuchiara ML, Davies JL, Matthews KRW (2013). Defining “research” in the US and EU: contrast of Sherley v. Sebelius and Brüstle v. Greenpeace rulings. Stem Cell Rev Rep.

[CR6] Deglincerti A, Croft GF, Pietila LN, Zernicka-Goetz M, Siggia ED, Brivanlou AH (2016). Self-organization of the in vitro attached human embryo. Nature.

[CR7] Department of Health and Social Security (1984). Report of the committee of inquiry into human fertilisation and embryology.

[CR8] Doerflinger RM (1999). The ethics of funding embryonic stem cell research: a Catholic viewpoint. Kennedy Inst Ethics J.

[CR9] Gu Z, Guo J, Wang H, Wen Y, Gu Q (2020). Bioengineered microenvironment to culture early embryos. Cell Prolif.

[CR11] Hurlbut JB (2017). Revisiting the Warnock rule. Nat Biotechnol.

[CR12] Hyun I, Wilkerson A, Johnston J (2016). Revisit the 14-day rule. Nature.

[CR13] Hyun I, Bredenoord AL, Briscoe J, Klipstein S, Tan T (2021). Human embryo research beyond the primitive streak: It is time to revisit the “14-day limit”. Science.

[CR10] International Society for Stem Cell Research (2021) Guidelines for stem cell research and clinical translation. https://www.isscr.org/policy/guidelines-for-stem-cell-research-and-clinical-translation

[CR14] Li Z, Di J (2021) Foreword: prevention and control of birth defects in China: achievements and challenges. China CDC Weekly. http://weekly.chinacdc.cn/en/article/doi/10.46234/ccdcw2021.191 10.46234/ccdcw2021.191 PMC8441183 34594987

[CR15] Ma H, Zhai J, Wan H, Jiang X (2019). In vitro culture of cynomolgus monkey embryos beyond early gastrulation. Science.

[CR16] Matthews KR, Moralí D (2020). National human embryo and embryoid research policies: a survey of 22 top research-intensive countries. Regen Med.

[CR17] McCully S (2021). The time has come to extend the 14-day limit. J Med Ethics.

[CR18] Niu Y (2019). Dissecting primate early post-implantation development using long-term in vitro embryo culture. Science.

[CR19] Peng Y, Huang X, Zhou Qi (2020). Ethical and policy considerations for human embryo and stem cell research in China. Cell Stem Cell.

[CR20] Poplawski N, Gillett G (1991). Ethics and embryos. J Med Ethics.

[CR21] Regalado A (2021) Scientists plan to drop the 14-day embryo rule, a key limit on stem cell research. https://www.technologyreview.com/2021/03/16/1020879/scientists-14-day-limit-stem-cell-human-embryo-research/

[CR22] Reichlin M (1997). The argument from potential: a reappraisal. Bioethics.

[CR23] Shahbazi MN, Jedrusik A, Vuoristo S, Recher G (2016). Self-organization of the human embryo in the absence of maternal tissues. Nat Cell Biol.

[CR24] Sozen B (2021). Reconstructing aspects of human embryogenesis with pluripotent stem cells. Nat Commun.

[CR25] Wadman M (2021). United Kingdom moves to prevent birth defects. Science.

[CR26] Weatherbee BA (2021). Modeling human embryo development with embryonic and extra-embryonic stem cells. Dev Biol.

[CR27] Zhang JY (2012). The cosmopolitanization of science: stem cell governance in China.

